# Identification and Characterization of Novel Malto-Oligosaccharide-Forming Amylase AmyCf from *Cystobacter* sp. Strain CF23

**DOI:** 10.3390/foods12183487

**Published:** 2023-09-19

**Authors:** Jihong Wang, Lei Zhang, Peiwen Wang, Jinhui Lei, Lingli Zhong, Lei Zhan, Xianfeng Ye, Yan Huang, Xue Luo, Zhongli Cui, Zhoukun Li

**Affiliations:** 1 Key Laboratory of Agricultural Environmental Microbiology, Ministry of Agriculture, College of Life Science, Nanjing Agricultural University, Nanjing 210095, China; 2023216020@stu.njau.edu.cn (J.W.); 2020216016@stu.njau.edu.cn (L.Z.); 2022816142@stu.njau.edu.cn (J.L.); 2022216012@stu.njau.edu.cn (L.Z.); 10121101@stu.njau.edu.cn (L.Z.); yxf@njau.edu.cn (X.Y.); huangyan@njau.edu.cn (Y.H.);; 2 Department of Prevention and Control of Infectious Disease, Henan Centers for Disease and Prevention, No. 105, Nong Ye Dong Lu, Zhengzhou 450016, China

**Keywords:** Malto-oligosaccharides, *Cystobacter*, gelatinized starch, enzymatic conversion, raw starch

## Abstract

Malto-oligosaccharides (MOSs) from starch conversion is advantageous for food and pharmaceutical applications. In this study, an efficient malto-oligosaccharide-forming α-amylase AmyCf was identified from myxobacter *Cystobacter* sp. strain CF23. AmyCf is composed of 417 amino acids with N-terminal 41 amino acids as the signal peptide, and conserved glycoside hydrolase family 13 (GH13) catalytic module and predicted C-terminal domain with β-sheet structure are also identified. Phylogenetic and functional analysis demonstrated that AmyCf is a novel member of GH13_6 subfamily. The special activity of AmyCf toward soluble starch and raw wheat starch is 9249 U/mg and 11 U/mg, respectively. AmyCf has broad substrate specificity toward different types of starches without requiring Ca^2+^. Under ideal circumstances of 60 °C and pH 7.0, AmyCf hydrolyzes gelatinized starch into maltose and maltotriose and maltotetraose as the main hydrolytic products with more than 80% purity, while maltose and maltotriose are mainly produced from the hydrolysis of raw wheat starch with more than 95% purity. The potential applicability of AmyCf in starch processing is highlighted by its capacity to convert gelatinized starch and raw starch granules into MOSs. This enzymatic conversion technique shows promise for the low-temperature enzymatic conversion of raw starch.

## 1. Introduction

Malto-oligosaccharides (MOSs) are linear oligosaccharides consisting of around 3–10 α-D-glucopyranosyl units linked by α-1,4 glycosidic linkages. As functional ingredients, MOSs and their derivatives have been widely used in the food, pharmaceutical and other industries, which can be used as sweeteners, preservatives, bulking agents, thickeners, humectants and prebiotics [1]. In the context of food baking, oligosaccharides have an affinity for the surface or interior of starch granules, and this interaction leads to an elevation in both peak viscosity and gelatinization temperature of the starch [2]. MOSs can be prepared by enzymatic hydrolysis of starch via specific enzymes, and Malto-oligosaccharides-forming amylases, glycosyl transferases and cyclodextrin-hydrolyzing enzyme are suggested as excellent candidates, such as maltogenic amylase (G2) from *Corallococcus* sp. EGB [3], maltotriose-producing amylase (G3) from *Kitasatospora* [4], maltotetraose-producing amylase (G4) from *Bacillus halodurans* MS-2–5 [5], maltopentaose-forming amylase (G5) from *Bacillus stearothermophilus* [6], maltohexaose-forming amylase [7] and GH77 4-α-glucanotransferase CcGtase from *Corallococcus* sp. EGB [8]. To achieve efficient enzymatic hydrolyzation, pre-treatment of raw starch under higher temperature is needed to destroy the internal ordered structure of starch particles. This requires the amylases to exhibit higher thermostabilities from the one-step hydrolysis process [9]. However, enzyme activities of most of these enzymes are gradually lost at temperatures above 60 °C, suggesting poor thermostabilities. Hence, the utilization of raw-starch-digesting enzymes (RSDEs) to hydrolyze raw starch at temperatures below gelatinization is a viable approach for the creation of modified starches. 

Thus far, RSDEs originating from bacteria, filamentous fungus, yeasts and plants have demonstrated effective activity toward raw starch [10]. RSDEs have been reported to be members of α-amylase, amylopullulanase, cyclomaltodextrin glucanotransferase, β-amylase and glucoamylase [11]. Among them, α-amylase is the main representative of family GH13 from the CAZy database [12], which are composed of the main catalytic (β/α)_8_-barrel domain, a small domain protruding out of the barrel as a longer loop between the strand β3 and helix α3 and a C-terminal domain [13]. Even these amylolytic enzymes share conserved structural feathers, with only approximately 10% of amylolytic enzymes possessing the capacity to digest raw starch, which may be attributed to the insoluble surface and hierarchical interior structures that prevent access-binding and subsequent amylase activity [11]. Starch-binding domains (SBD) from some RSDEs promote the substrate attachment at the solid–fluid interface, which may support enzymatic degradation of raw starch [14], and deletion of SBD from RSDEs results in loss of digestibility on raw starch [11,15]. In the sequence-based classification, individual types of SBD have been classified into fifteen CBM families [16]. The domain functions of the family GH13 members in the hydrolysis of raw starch need further investigation. 

To date, RSDEs have been evaluated in production of ethanol, porous starch, cyclodextrin and MOSs. Among the enzymes under consideration, α-1,4-amylase AmyZ1 from *Pontibacillus* sp. ZY possesses a significantly elevated specific activity of 12, 621 U/mg, enabling the hydrolysis of raw corn starch into G1 (15.4%), G2 (27.2%) and G3 (32.7%) [17]. Furthermore, AmyM from *Corallococcus* sp. EGB hydrolyzes raw starch into G2 (41.3%), G3 (30.5%) and G4 (10.2%) [18]. These findings establish them as excellent candidates for raw starch conversion. Otherwise, commercial products regarding raw starch hydrolysis, like Novozyme 50009 and STARGEN™ (DuPont), have been developed. These products have been applied for raw starch saccharification with the aim to hydrolyze starch into glucose [19]. Hence, for MOSs preparation, it is imperative to investigate novel MOSs-forming amylases with efficient hydrolysis ability and substrate selectivity. 

Myxobacteria are microbial factories for the discovery of novel metabolites and lytic enzymes, and abundant of enzymes associated with starch, chitin, pectin, cellulose, β-glucan and xylan conversion have been identified, including hydrolases [18,20,21,22], and polysaccharide monooxygenases [23], and part of them have been applied in preparation of functional glucan [24]. However, only maltohexaose-forming α-amylase AmyM from myxobacteria has been identified with activity toward raw starch granules [18], indicating the potential feasibility for exploration of valuable RSDEs from myxobacteria. In this study, a novel MOSs-producing amylase AmyCf was identified from myxobacteria *Cystobacter* sp. strain CF23, which exhibited sequence novelty compared to the reported amylases. AmyCf showed efficient hydrolysis of soluble starch into G2 and G3 and G4, and also exhibited activity toward raw starch with G2 and G3 as the main products. The objective of this study was to characterize the novel GH13 MOSs-producing amylase from myxobacteria and its potential application in MOSs preparation. 

## 2. Materials and Methods

### 2.1. Materials

The cloning of the α-amylase gene was performed using *Cystobacter* sp. strain CF23 as the source. The host strains utilized for gene cloning and protein expression were *Escherichia coli* DH5, *Escherichia coli* BL21 and *Pichia pastoris* GS115 (Invitrogen Corporation, Shanghai, China). The vector pEFαA was used for protein expression [3]. The starches obtained for the study were procured from Changhong Potato Starch Co., Ltd. (located in Hebei, China) and Yuanye Biotechnology Co., Ltd. (based in Shanghai, China). The Malto-oligosaccharides used in this study were sourced from Aladdin Co., Ltd. (Shanghai, China). The chemicals and reagents employed in this work were sourced from Sinopharm Chemical Reagent Co., Ltd. (located in Shanghai, China) and were of analytical grade. 

### 2.2. Plasmid Construction and Gene Expression

The total genomic DNA extracted from *Cystobacter* sp. strain CF23 was used as a template for PCR amplification of the predicted α-amylase using the primers AmyCf-F5′-CCGCTCGAGATGGGGCCGCTGGATG-3′ and AmyCf-R 5′-GCTCTAGACTTCGTCCAGACGGCG-3′. The PCR amplifications were performed using PrimeSTAR HS DNA Polymerase (Takara, Japan), and the obtained PCR fragments were digested with XhoI and XbaI, following by inserting into the same digested sites of pEFαA to obtain the pEFαA-amyCf plasmid. Then, we transformed *P. pastoris* GS115 with the pEFαA-amyCf-based plasmid and isolated the positive transformants which could grow on YPD plates containing 100 mg/L zeocin (Invitrogen, R25001). Inducible expression of the amylase AmyCf in *P. pastoris* was performed as described before [8]. Briefly, isolated transformants were grown in 50 mL YPD medium (1% yeast extract, 2% peptone and 2% glucose) at 30 °C and 200 rpm for 24 h, followed by transferring cells into 100 mL of BMMY medium (1% yeast extract, 2% peptone, 1% glycerol, 1.34% YNB, 0.1 mol/L phosphate buffer and 0.04 mg/L biotin). Methanol induction was conducted after the first 12 h of incubation, followed by feeding of 0.5% (*v*/*v*) methanol at 12 h intervals for the whole 96 h fermentation period [25]. The medium containing *P. pastoris* GS115 cells was cultured at 30 °C and 200 rpm for 4 days, the cell-free supernatant was collected and assayed for enzyme activity. The protein content was determined using the Bradford [7]. 

### 2.3. Enzyme Assay

The enzymatic activities of AmyCf toward soluble starch and raw starch were assessed using the dinitrosalicylic acid (DNS) technique by determining the quantity of released reducing sugars. The enzymatic reaction of AmyCf toward soluble starch or raw starch was performed at 60 °C or 50 °C in 50 mM Tris-HCl buffer (pH 7.0), respectively. One unit of α-amylase activity was defined as the amount of enzyme required to release 1 μmol of glucose under the standard assay conditions. 

### 2.4. Biochemical Properties of the α-Amylase AmyCf

To determine the optimal reaction temperature of AmyCf toward soluble starch or raw wheat starch (*w*/*v*, 1.0%), reaction mixtures containing AmyCf and substrates were incubated at temperatures between 30 and 70 °C for 30 min. The thermal stability experiments were performed by incubating the produced AmyCf at various temperatures, and afterwards evaluating the remaining activity under standardized conditions. The determination of the best reaction pH was conducted under normal conditions by employing multiple buffers with varying pH values. Additionally, the pH stabilities of AmyCf were assessed by subjecting the proteins at 4 °C in different buffers for an incubation of 24 h, followed by determining the residual activity. All of the aforementioned processes were conducted in the absence of Ca^2+^. In order to assess the impact of metal salts on the activity of AmyCf, the enzyme solution was subjected to pre-treatment with 1 mM EDTA at a temperature of 4 °C for 24 h. Subsequently, the sample was dialyzed against a 50 mM Tris-HCl buffer with a pH 7.0 to eliminate any remaining EDTA. The effects of metal ions on the activity of AmyCf were carried out by the addition of various metals with 1 and 5 mM concentration (K^+^, Na^+^, Cu^2+^, Ca^2+^, Mn^2+^, Ni^2+^, Mg^2+^, Zn^2+^, Co^2+^, Ni^2+^, Ba^2+^, Fe^3+^, Cr^3+^) under standard condition. The residual activity was assessed as described above. Activity without any additive was regarded as 100%. 

The substrate specificity of AmyCf was determined by measuring the hydrolysis activity toward various gelatinized starches (soluble starch, starch from potato, amylopectin from potato, amylose from potato, glycogen, α-clycodextrin, pullulan, 1.0%, *w*/*v*) at 60 °C and pH 7.0 (Tris-HCl buffer, 50 mM). Otherwise, the hydrolytic activity of AmyCf toward raw starches (starches from potato, corn, wheat and cassava, soluble starch, amylose, amylopectin, 1.0%, *w*/*v*) was also measured at 50 °C in 50 mM Tris-HCl buffer (pH 7.0). 

### 2.5. Analysis of the Hydrolyzed Products

The prepared AmyCf was incubated with different starch samples (1.0%, *w*/*v*), or maltotetraose (G4), maltopentaose (G5) and maltohexaose (G6) in 50 mM Tris-HCl (pH 7.0) at 60 °C for gelatinized starch or 50 °C for raw starch. In order to ascertain the products distribution from the hydrolysis of starches, the supernatant of the reaction mixture was obtained subsequent to centrifugation. Subsequently, the supernatant was subjected to heat treatment at 100 °C for 10 min to inactivate the enzyme. Thin-layer chromatography (TLC) analysis was performed to examine the composition of the products. For TLC analysis, the prepared hydrolysates were spotted onto the silica gel 60 plates (Merck, Germany) and separated using n-butanol/methanol/H_2_O (8:4:3, *v*/*v*/*v*) as the solvent system [7]. The visualization of the spots on the plates corresponding to the sites of MOSs was achieved by applying a solution of sulfuric acid-methanol (1:1, *v*/*v*) with a heating treatment at 90 °C for 10 min. Malt-oligosaccharide with degrees of polymerization (DP) ranging from 1 to 7 were used as the standards. 

### 2.6. Sequence Analysis

The conserved domains and catalytic residues of AmyCf were analyzed by Conserved Domain Search via the Conserved Domain Database (CDD) of the National Center for Biotechnology Information (NCBI) [26]. A total of 241 representative members belonging to 47 GH13 subfamilies (from GH_1 to GH_47) were obtained from the CAZy database (http://www.cazy.org/ (accessed on 26 August 2023)), and sequence comparison and phylogenetic analysis of AmyCf with other 241 members of the family GH13 α-amylases were analyzed by MEGA 11. Based on the classification of subfamily, the alignment of the amino acid sequences of AmyCf with the members of assigned GH13 subfamily was performed using ClustalW version 2.0. The signal peptide was predicted using the SignalP 5.0 (https://services.healthtech.dtu.dk/services/SignalP-5.0/ (accessed on 12 August 2023)), and the isoelectric point (pI) and molecular mass were determined by the Compute pI/Mw tool (https://web.expasy.org/compute_pi/ (accessed on 12 August 2023)). The protein structure of AmyCf was predicted by AlphaFold algorithm [27]. 

### 2.7. Statistical Analysis

All data and experiments were presented and performed in triplicate, and mean values were designed with standard deviations. The data were analyzed utilizing Duncan’s multiple range test, which were operated in SPSS software (ver. 22.0; IBM Corp, Armonk, NY, USA). 

### 2.8. The Accession Number of the Nucleotide Sequence

The nucleotide sequence corresponding to the amylase gene amyCf, which was obtained from *Cystobacter* sp. strain CF23, has been officially recorded in the GenBank database with the accession number OR405308. 

## 3. Results and Discussion 

### 3.1. Identification of a Novel α-Amylase AmyCf from Cystobacter sp. Strain CF23

By genome comparison analysis, a potential Malto-oligosaccharides-forming amylase was identified from *Cystobacter* sp. strain CF23 and accordingly named AmyCf, which is composed of 417 amino acid residues. Sequence analysis showed that AmyCf contains a N-terminal putative signal peptide, GH13 catalytic module and C-terminal β-sheet domain, which shared the general structural feature with family GH13 α-amylase [13]. The typical SBD, which is classified as a carbohydrate-binding module (CBM) within the CAZy database, is absent in AmyCf. The BLASTP analysis revealed that AmyCf shares high sequence identity (90%) with the α-amylase derived from *Cystobacter* fuscus. Subsequently, the α-amylases originating from *Archangium* and *Stigmatella hybrida* displayed a sequence identity of 84%. However, these proteins from myxobacteria are annotated as predicted α-amylase family without biochemical characterization. 

**Figure 1:**
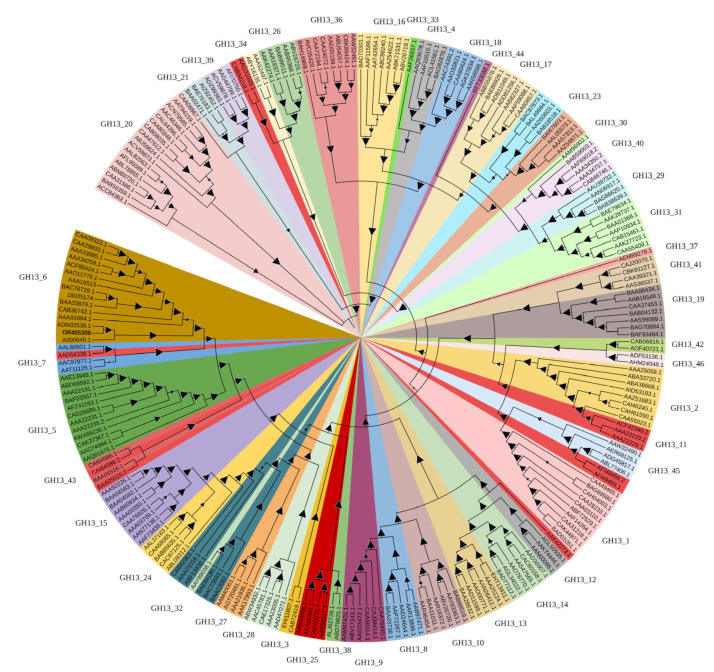
Evolutionary tree of α-amylases of GH13 family. The tree illustrates the relatedness of 241 members from 47 different GH13 subfamilies (from GH_1 to GH_47). The tree is based on alignment of complete α-amylase sequences extracted from the CAZY database with a corresponding GenBank number linking with the NCBI database. The individual GH13 subfamilies are distinguished from each other by different colors. The tree was calculated as a maximum-likelihood tree using the MEGA 11 software [37], http://www.megasoftware.net/ (accessed on 15 September 2023)) applying default programmed parameters, which was further displayed with the program iTOL+ (http://itol.embl.de/ (accessed on 15 September 2023)).

Currently, α-amylase enzyme specificity within the CAZy database can be found unambiguously in the families GH13, GH57 and GH119; the family GH13 is considered to be the main α-amylase family first proposed in the CAZy system [12]. In general, members of GH13 α-amylases are three-domain proteins, and AmyCf displays the consistent structural feature, indicating the classification of AmyCf as a member of the family GH13. Up to now, GH13 was broken up into 46 subfamilies from CAZy database, and the members of GH13 subfamily share a more recent evolutionary ancestor. To further identify the subfamily assignment of AmyCf, phylogenetic analysis of AmyCf with the members of 46 subfamilies family of GH13 from CAZy database was performed. As shown in Figure 1, AmyCf was assigned to subfamily GH13_6, which has the consistent evolutionary lines. Up to now, 18 Characterized α-amylases were reported from the CAZy database, including 2 α-amylases from bacteria, 15 α-amylases from plants and 1 unclassified member. Among them, are the maltohexaose-forming α-amylase AmyM from *Corallococcus* sp. EGB [8] and the possible G1 + G2-producing α-amylase Amy1 from *Massilia timonae* CTI-57 [28], which show sequences identities of 42% and 51% with AmyCf, respectively. Otherwise, AmyCf has low sequence identity (<35%) with other 16 members of subfamily GH13_6. According to the CAZy nomenclature, the α-amylases from plants and archaea have been assigned the subfamily number GH13_6 and GH13_7, respectively [29], and the cluster of archaeal α-amylases shares the branch with that of plant counterparts [30,31], indicating the possible evolutionary relatedness. Within the subfamily GH13_6, α-amylases from wheat and rice display an important function in starch granules hydrolysis during seed development [31], the same subfamily classification reflects the possibility of raw starch hydrolytic activity of AmyCf. 

In our previous studies, maltohexaose-forming α-amylase AmyM [7], α-amylase AmyC [32], maltogenic amylase CoMA [9] and G2 + G3-producing amylase AmyAc [33] have been identified from myxobacteria *Corallococcus* and *Archangium*. AmyCf from *Cystobacter* shares low sequence identity (<30%) with these enzymes except for AmyM (42%), and AmyCf is the first identified α-amylase from *Cystobacter*. Phylogenetic analysis showed that AmyCf and amylase AmyM from myxobacteria form an independent branch, which is separated from the known MOS-producing amylases from other microbes (Figure S1a). Especially, even AmyCf and maltotetraose-forming amylase AmyP from *Pseudomonas stutzeri* MO-19 [32] share similar evolutionary lines, while different evolutionary branches. Multiple sequence alignment and domain prediction showed that AmyCf contains the conserved catalytic triad Asp174-Glu199-Asp276, demonstrating the conserved feathers of glucoside hydrolase (Figure S1b). Members of family GH13 are commonly composed of three domains: N-terminal catalytic (β/α)_8_-barrel domain, irregular domain between the strand β3 and helix α3, C-terminal domain [13]. Structural prediction showed that AmyCf and the three representative members of subfamily GH13_6 display the consistent three-domain composition (Figure S2) [34], demonstrating the typical feathers of the members of GH13 family. 

Myxobacteria have been found to inhabit the dung of herbivores, dead and decayed woods and plant materials, and possess the ability to decompose the polysaccharide-like cellulose and starch [35]. Meantime, myxobacteria could secrete an abundance of extracellular enzymes with the ability to degrade biomacromolecules or even microorganisms [36], indicating that myxobacteria are valuable resources for the discovery of distinct industrial enzymes with potential catalytic diversity.

### 3.2. Expression of the Recombinant α-Amylase AmyCf in P. pastoris

**Figure 2:**
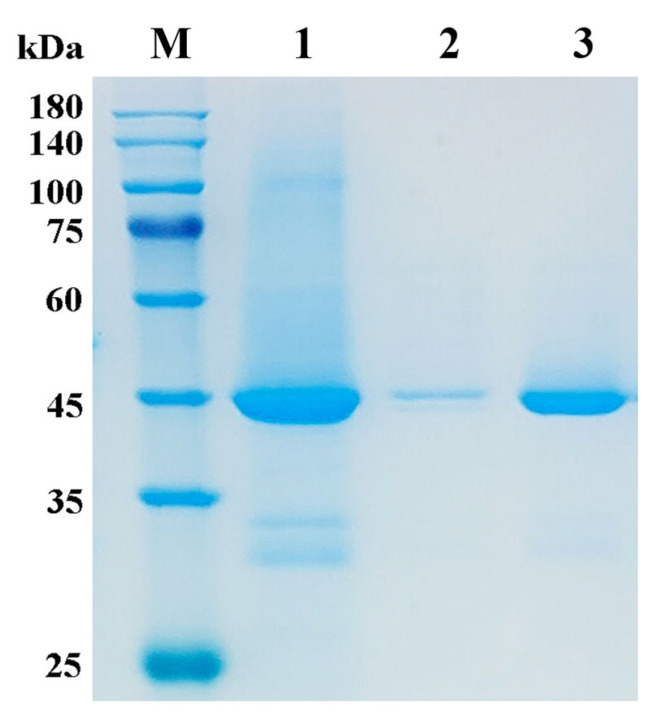
SDS-PAGE analysis of the prepared α-amylase AmyCf from *P. pastoris* GS115. Lane M, protein molecular weight marker; Lane 1–3 indicate the fermentation supernatant of *P. pastoris* GS115 containing pEFαA-amyCf, redissolution fraction from AS precipitation of the supernatant, ultrafiltration concentration of the redissolution fraction; AS: ammonium sulfate.

Considering the failing expression of AmyCf in *E. coli* with pET29a as the expression vector, the recombinant plasmid pEFαA harboring the amyCf gene was constructed and introduced into *P. pastoris* GS115 by electroporation. After selection, clones containing the amyCf gene were identified by PCR. After culturing in a shake flask at 30 °C with the induction of 0.5% methanol, AmyCf was successfully expressed with an apparent molecular mass of 42.8 kDa and 277.7 mg/L (Figure 2), indicating the powerful tool of *P. pastoris* for efficient expression of industrial enzyme. Glycosylation of the produced proteins in *P. pastoris* has been well recognized as an inherent alteration within the eukaryotic expression system [38]. In our previous study, we identified that AmyM from *Corallococcus*, which shares 45% sequence identity with AmyCf, exhibits 59.6% glycosylation content after extracellular expression in *P. pastoris* GS115 [3], whereas, glycosylation with greater apparent molecular mass was not observed from AmyCf. 

### 3.3. Characterization of the Recombinant α-Amylase AmyCf

**Figure 3:**
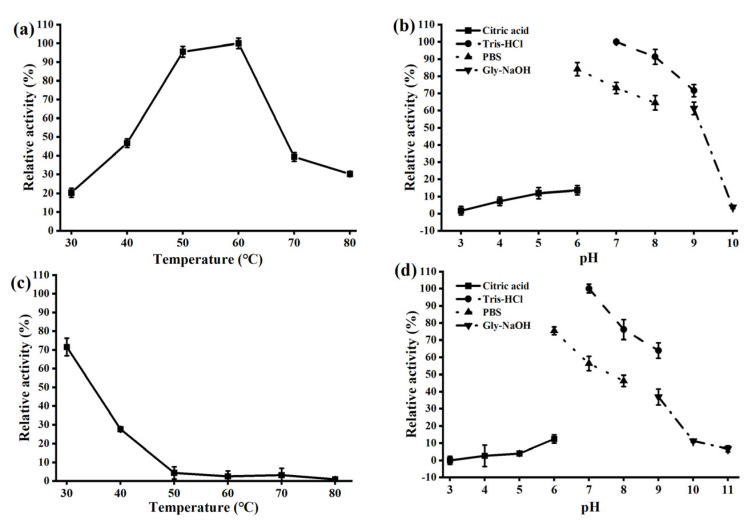
Effects of temperature and pH on activity and stability of AmyCf toward gelatinized starch. The optimal temperature (**a**) and optimal pH (**b**), thermostability (**c**) and pH stability (**d**) of AmyCf were analyzed using gelatinized soluble starch as the substrate. The data represent the averages from triplicate experiments. The test conditions were provided in the part of 2.4.

**Figure 4:**
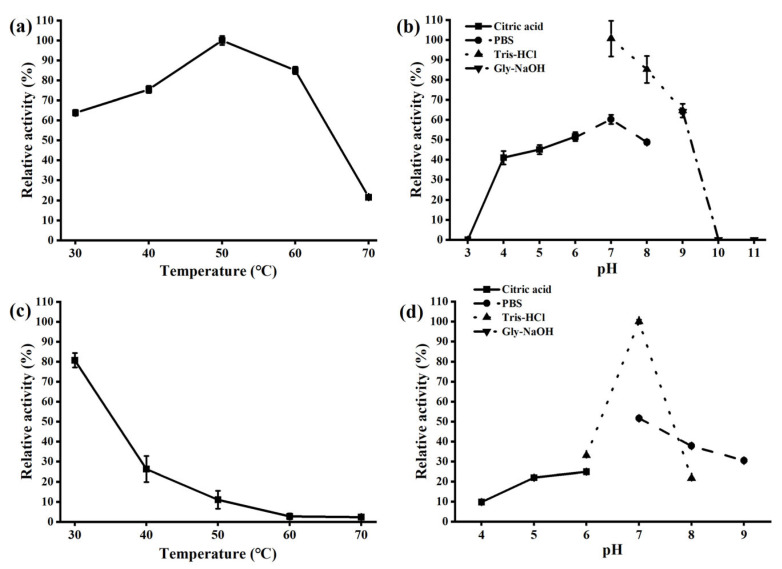
Effects of temperature and pH on activity and stability of AmyCf toward raw starch. The optimal temperature (**a**) and optimal pH (**b**), thermostability (**c**) and pH stability (**d**) of AmyCf were analyzed using wheat raw starch granules as the substrate. The data represent the averages from triplicate experiments. The test conditions were provided in the part of 2.4.

Biochemical characterization showed that AmyCf displays efficient activity toward gelatinized starch and also raw starch without heating treatment. Using soluble starch as the optimal substrate, AmyCf displayed optimal activity at 60 °C and pH 7.0 (Figure 3a,b), and significant thermal instability (Figure 3c) and moderate pH stability (Figure 3d) were also observed. The specific activity of AmyCf toward gelatinized soluble starch was determined as 9249 U/mg. To determine whether AmyCf is active against raw starch, we used raw wheat starch as the substrate. AmyCf displayed optimal activity at 50 °C and pH 7.0 (Figure 4a,b), and showed thermal instability and pH stability at pH 7−10 (Figure 4c,d). Under the optimal conditions, AmyCf had a specific activity of 11 U/mg using raw wheat starch as the substrate. Commonly, most of the reported GHs from myxobacteria are active at an intermediate temperature and keep thermal ability under 50 °C [3,7], similar to most maltooligosaccharide-producing amylases. Up to now, except for AmyM [18], AmyCf is the second reported raw-starch-digesting enzyme identified from myxobacteria. 

The effects of various metal ions on the activity of the recombinant α-amylase AmyCf toward soluble starch and raw starch were also investigated (Table 1). Most tested metal ions, like Cu^2+^, Mg^2+^, Mn^2+^, Ba^2+^, Zn^2+^, Fe^3+^ and Cr^3+^ showed negative effects on the activity of AmyCf toward gelatinized or raw starch samples. Co^2+^ commonly exhibited no effects on activity of amylase [39], while 1 mM Co^2+^ promotes the activity of AmyCf toward gelatinized and raw starch by 46% and 16%, respectively. Ca^2+^ is an important additive for the stability and activity of most α-amylases in industrial conditions [4]. As expected, 1 mM Ca^2+^ promoted the activity of AmyCf by 46%, while Ca^2+^ at 1 mM had no obvious effect on the activity of AmyCf toward raw starch. These results indicated that the activity of AmyCf toward gelatinized starch is independent of Ca^2+^, but not for raw starch. AmyCf from *Cystobacter* shares about 42% sequence identify with amylase AmyM from *Corallococcus*, while starch hydrolysis of AmyM was independent on Ca^2+^ [7]. Meantime, the activities of malto-oligosaccharide-producing amylase AmyAc from *Archangium* and maltogenic amylase CoMA from *Corallococcus* were inhibited by 30−40% in the presence of 5 mM Ca^2+^ [3,32], which are different from AmyCf. Otherwise, changes occurred in a native starch granules–water mixture during heating include gelatinization are associated with swelling and amylose leaching and partial granule disruption, which results in the formation of a starch paste [40]. The different performances of Ca^2+^−dependences toward gelatinized and raw starch samples indicate that AmyCf exhibits different catalytic behavior toward starch chains with various conformation. 

### 3.4. Substrate Specificity of Recombinant α-Amylase AmyCf

Substrate specificity of AmyCf was conducted by employing different starch samples as the substrates for the enzymatic reaction (as shown in Table 2). When using gelatinized starch as the substrates, AmyCf displayed the highest activity toward soluble starch, followed by potato starch, amylopectin and amylose, comparatively lower degree of hydrolysis. In contrast, AmyCf exhibited the ability to enzymatically break down several types of raw starches (A, B, and C types) at 50 °C. Notably, AmyCf displayed a preference for the hydrolysis of wheat raw starch. A type wheat starch granules is composed of 25% amylose and 75% amylopectin, and the amylopectin generally exhibits a smooth polymodal distribution of branch chains with the peak maxima at DP 11–12 [41]. While the amylose of potato starch (B type) is relatively long and linear chains with few branches, amylopectin of potato starch is a heavily branched structure [42]. This structural basis may affect the hydrolytic activity of AmyCf by possible starch chain binding and recognition. Previous research showed that MOSs-forming amylases with the hydrolytic activity of raw starch exhibit positive effects on the bread quality and starch digestibility of normal and waxy wheat [18,43,44]. The favored hydrolysis of raw wheat starch may enable AmyCf as a potential bread improver. 

### 3.5. Action Pattern of Recombinant α-Amylase AmyCf toward Starch and MOSs

**Figure 5:**
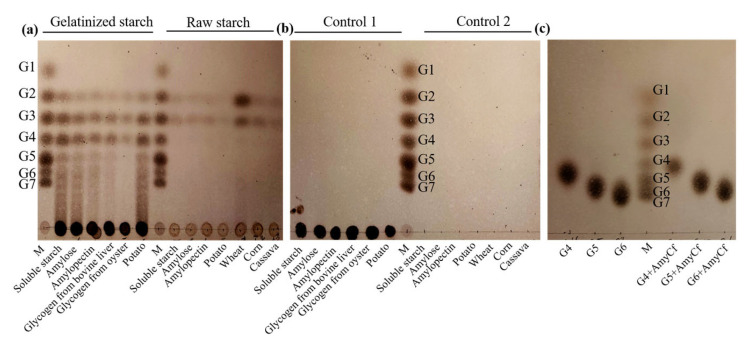
TLC analysis of the hydrolysis products of AmyCf toward starch and MOSs. The α-amylase AmyCf was incubated with gelatinized starch in Tris-HCl buffer (50 mM, pH 7.0) at 60 °C, and the hydrolysis products were analyzed by TLC ((**a**), Gelatinized starch), and the heat-inactive enzyme was used as control ((**b**), Control 1). The α-amylase AmyCf was also incubated with raw starch in Tris-HCl buffer (50 mM, pH 7.0) at 50 °C, and the hydrolysis products were analyzed by TLC ((**a**), Raw starch), and the heat-inactive enzyme was used as control ((**b**), Control 2). The used starch samples are soluble starch, potato starch, wheat starch, cassava starch, corn starch, glycogen, amylose and amylopectin. The α-amylase AmyCf was incubated with maltotetraose (G4), maltopentaose (G5) and maltohexaose (G6) at 60 °C and pH 7.0 for 30 min, and the hydrolysis products were analyzed by TLC (**c**).

To analyze the product distribution of AmyCf from hydrolysis of different substrates, AmyCf was incubated with various MOSs and starch samples. As shown in Figure 5, AmyCf hydrolyzed different gelatinized starch samples and glycogen into maltose (G2) and maltotriose (G3) and maltotetraose (G4) as the main end-products with more than 80% composition, along with slight oligosaccharides with DP value more than 4 (Figure 5a). However, G2 and G3 were mainly produced from the hydrolysis of raw starch with more than 95% purity. During the hydrolytic process, no glucose was detected. This differences in product distribution were also observed from the α-1,4-amylase AmyZ1 from *Pontibacillus* sp. ZY [17] and maltohexaose-forming α-amylase AmyM from *Corallococcus* sp. EGB [18], indicating that these amylases with the hydrolytic activity of raw starch display diverse catalytic properties toward starch chains of granules or gelatinized starch after heating treatment. Otherwise, no hydrolytic or glucanotransferase activity was observed from G4, G5 and G6 (Figure 5c), indicating the strict substrate selectivity of AmyCf. In our previous study, we identified that AmyAc from *Archangium* hydrolyzes G4 and G5 into G2 + G2 and G2 + G3, respectively, resulting the accumulation of G2 and G3 as the main products [33]. No action of AmyCf toward G5 and G6 indicates that AmyCf may bind starch chains with DP values more than 9, resulting in the production of G2, G3, and G4. However, further evidence is required to support this hypothesis. Otherwise, AmyCf was assigned as a member of the subfamily GH13_6 in the CAZy database by sequential evolution analysis, which shares 42% and 51% sequences identities with maltohexaose-forming α-amylase AmyM from *Corallococcus* sp. EGB [7] and the possible G1 + G2-producing α-amylase Amy1 from *Massilia timonae* CTI-57 [28]. However, AmyM hydrolyzed gelatinized starch into G6 as the main product, and Amy1 hydrolyzed soluble starch into G1 and G2. Combing the sequence identity and hydrolytic function, we deduced that AmyCf is a novel member of subfamily GH13_6. 

Commercial barley β-amylase and maltogenic amylase (Novozyme) have been employed for the purpose of maltose conversion. However, it is worth noting that their ability to hydrolyze raw starch is constrained. Otherwise, most of the reported amylases operate in an endo-lytic manner, a characteristic that influences the presence of glucose in the resulting products. However, feasibility of MOSs is dependent on their DP values [1]. The presence of glucose limits the health benefits due to the blood glucose response in humans [43]. The efficient conversion of starch with diverse states by AmyCf results in preparation of MOSs with high purity, suggesting that AmyCf exhibits product selectivity, hence demonstrating its potential superiority in various industrial applications. 

## 4. Conclusions

In this study, we identified a special Malto-oligosaccharides-producing amylase AmyCf from *Cystobacter* sp. strain CF23 with hydrolytic activity of raw starch, which shares low sequence identity (<45%) with the reported amylases. Under the optimal conditions, the special activities of AmyCf toward soluble starch and raw wheat starch are identified as 9249 U/mg and 11 U/mg, respectively. AmyCf effectively hydrolyzes gelatinized starch into G2, G3 and G4 without depending on Ca^2+^. It is noteworthy that AmyCf also displays the ability to hydrolyze raw starch into G2 and G3 with the absence of glucose. The uniform hydrolysis and diverse breakdown abilities makes AmyCf a highly viable choice for the enzymatic hydrolysis of starch into MOSs. Further research is warranted to explore the catalytic mechanism of AmyCf involved during hydrolysis processes, with particular emphasis on understanding the substrate selectivity. 

## Figures and Tables

**Table 1:** The effects of metal ions on the activity of α-amylase AmyCf.

The Type of Metal Ion	Soluble Starch Relative Activity (%)		Raw Wheat Starch Relative Activity (%)	
	**1 mM**	5 mM	1 mM	5 mM
Control	100.00 ± 1.27	100.00 ± 0.58	100.00 ± 2.08	100.00 ± 1.21
K^+^	101.31 ± 1.77	145.49 ± 1.84	93.18 ± 0.59	91.24 ± 0.07
Na^+^	101.31 ± 1.81	132.19 ± 1.84	100.79 ± 6.22	102.18 ± 0.31
Ca^2+^	146.43 ± 2.10	128.47 ± 2.00	104.56 ± 1.05	106.83 ± 0.53
Co^2+^	133.29 ± 2.59	142.12 ± 1.80	116.49 ± 1.18	72.99 ± 0.68
Cu^2+^	116.62 ± 2.34	86.39 ± 1.51	58.79 ± 0.39	14.93 ± 1.72
Mn^2+^	115.85 ± 2.42	87.24 ± 1.88	72.35 ± 0.74	47.15 ± 0.01
Zn^2+^	110.53 ± 2.03	63.51 ± 1.26	61.65 ± 0.69	29.78 ± 1.20
Ni^2+^	109.97 ± 0.14	109.11 ± 2.18	86.69 ± 0.14	52.35 ± 0.14
Fe^3+^	104.74 ± 2.30	58.37 ± 1.38	74.02 ± 0.21	40.52 ± 0.96
Cr^3+^	97.35 ± 2.31	49.95 ± 1.42	59.37 ± 0.58	44.77 ± 0.75
Ba^2+^	96.76 ± 2.22	63.39 ± 1.51	89.76 ± 0.19	82.93 ± 0.08
Mg^2+^	87.42 ± 2.30	77.31 ± 1.78	69.77 ± 0.14	61.45 ± 0.69

**Table 2:** Substrate specificities of α-amylase AmyCf toward various substrates.

Soluble Starch		Raw Wheat Starch	
Substrate	Relatively Activity (%)	Substrate	Relatively Activity (%)
Soluble starch	100.0 ± 0.13	Wheat	100.00 ± 0.36
Starch from potato	95.0 ± 0.13	Soluble starch	30.87 ± 0.09
Amylopectin from potato	86.6 ± 1.70	Amylose	27.06 ± 0.08
Amylose from potato	70.8 ± 0.30	Corn	26.56 ± 0.08
Glycogen	5.8 ± 1.66	Amylopectin	22.86 ± 0.07
α-Cyclodextrin	0.0	Cassava	16.76 ± 0.04
Pullulan	0.0	Starch from potato	16.03 ± 0.14

## Data Availability

The data used to support the findings of this study can be made available by the corresponding author upon request.

## Supplementary Materials

The following supporting information can be downloaded at: https://www.mdpi.com/article/10.3390/foods12183487/s1, Figure S1: The phylogenetic tree was constructed using the neighbor-joining method based on the amino acid sequence alignment of catalytic domain (a), and the multiple sequence alignment of AmyCf and representative α-amylases from subfamily GH13_6 (b). Red asterisks indicate the conserved catalytic amino acids, and the GenBank Numbers indicate the source of enzyme.; Figure S2: Three-dimensional structure of AmyCf modeled using the deep learning algorithm AlphaFold2.
