# Supplementary material for: Identification and Characterization of Novel Malto-Oligosaccharide-Forming Amylase AmyCf from Cystobacter sp. Strain CF23
Source: Foods. 2023 Sep 19;12(18):3487. doi: 10.3390/foods12183487 (PMC10528286; doi:10.3390/foods12183487)
Supplement: Supplementary file 1 [file foods-12-03487-s001.zip › foods-2586345-supplementary.pdf]

Supplementary Materials

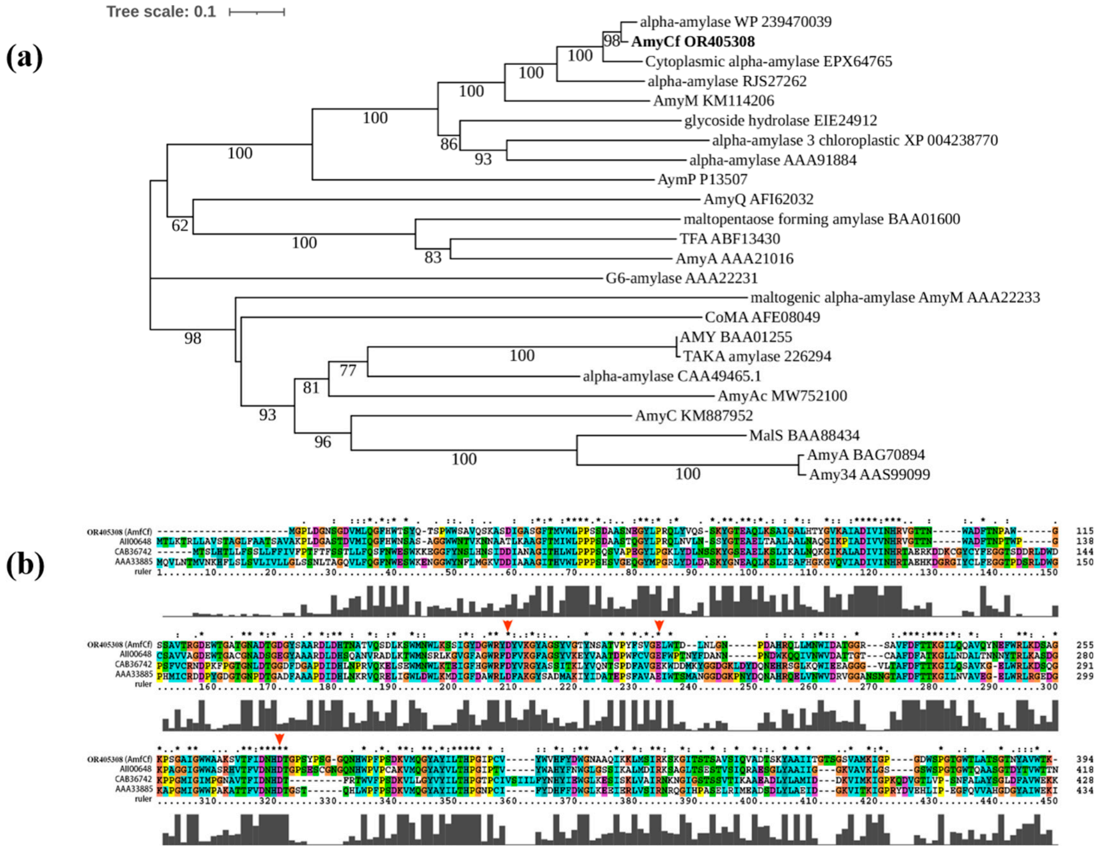

Figure S1. The phylogenetic tree was constructed using the neighbor-joining method based on the amino acid sequence alignment of catalytic domain (a), and the multiple sequence alignment of AmyCf and representative α-amylases from subfamily GH13_6 (b). Red asterisks indicate the conserved catalytic amino acids, and the GenBank Numbers indicate the source of enzyme.

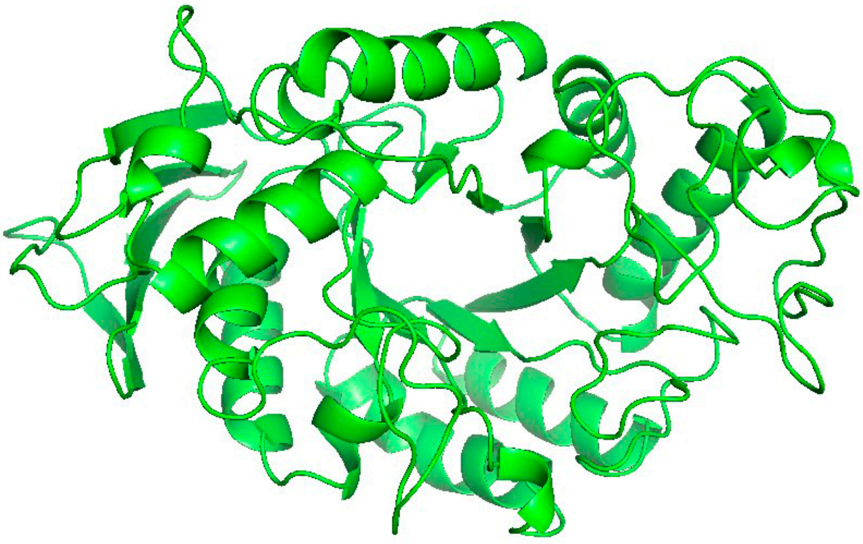

Figure S2. Three-dimensional structure of AmyCf modeled using the deep learning algorithm AlphaFold2.
